# Effects of Internal Partitions on Flow Field and Air Contaminant Distribution under Different Ventilation Modes

**DOI:** 10.3390/ijerph15112603

**Published:** 2018-11-21

**Authors:** Xiaoping Liu, Xiaojiao Wu, Linjing Chen, Rui Zhou

**Affiliations:** 1 School of Civil Engineering, Hefei University of Technology, Hefei 230009, China; liuxp@hfut.edu.cn (X.L.); wuxiaojiao16@126.com (X.W.); chenlinjing92@163.com (L.C.); 2 Department of Engineering Physics, Institute for Public Safety Research, Beijing Key Laboratory of City Integrated Emergency Response Science, Tsinghua University, Beijing 100084, China

**Keywords:** contaminant, airflow distribution, numerical simulation, concentration field

## Abstract

Based on frequently used internal partitions in offices, the effects of pollutant source characteristics and an internal partition on airflow and contaminant distribution under different ventilation modes are studied in this paper. The indoor flow field measurement is implemented in a 1:1 single environmental chamber under different ventilation patterns, and then the numerical model is established. The numerical method is verified and analyzed by comparing the measured and simulated results. According to the verification results, the numerical simulation is introduced to study the influence of different supply and return air mixes and pollutant source distributions on the flow field and diffusion performance with an internal partition. The indoor flow field and concentration distribution under different conditions are compared, and the discharge efficiency under different working conditions is analyzed. The results indicate that internal partitions have a greater influence on the down-supply up-return ventilation mode than the floor-supply up-return and top-supply down-return ventilation mode. Furthermore, if the room is zoned, the effect of source position is larger under the down-supply up-return ventilation mode than under the other two modes.

## 1. Introduction

In modern society, indoor air quality has become an important concern for public health due to the improper use of most building decoration materials and air-conditioning equipment. On average, people spend more than 90% of their time indoors [1]. Indoor air contaminants are closely related to people’s health: The contaminants will accumulate with poor ventilation, and the enclosed environment is more likely to deteriorate the indoor air quality [2,3].

Indoor air flow patterns under different ventilation modes are the dominant factors of contaminant diffusion and distribution, and pollutant source characteristics also play a key role in indoor contaminant distribution [4,5,6]. Many experts and scholars have studied indoor ventilation and contaminant distribution characteristics in a single room. Yang et al. analyzed the exhaust effectiveness of volatile organic compounds (VOCs) in an office in displacement and mixing ventilation based on a pollutant dispersion simulation model [7]. Li et al. deduced an analytic equation of indoor contaminant distribution by optimizing the single-chamber air supply model and selecting the appropriate pollutant dispersion model and initial conditions, and validated the equation based on the experimental data [8]. Zhao et al. studied the diffusion characteristics of particulate matter (PM) in an independent room, which revealed sedimentation characteristics of particulate pollutants under gravity by employing a three-dimensional drift flux model with the depositional boundary condition of the wall [9]. The results show that ventilation pattern, particle source position, and air exchange rate can affect the particle distribution in a room. Yang et al. executed a numerical simulation of indoor PM diffusion characteristics under top-supply, side-supply, and down-supply airflow in a single room, and compared the experimental data with the data obtained by Murakami et al. under the same air supply mode, which verified that they matched well [10,11]. The results proved that the down-supply airflow pattern is better for the removal of indoor particles, but with a potential re-entrainment problem. These studies provide a solid basis on the study of airflow patterns and pollutant distributions in a single room.

In addition to a general single room, indoor air quality in multiple rooms or multiple zones in a building under different ventilation strategies has also gained increasing attention [12]. For large open offices in current office buildings, the whole space is often zoned into an individual office environment by introducing internal partitions. Such partitions or the presence of furniture, equipment, and other indoor obstacles will lead to indoor airflow obstruction, steering, and other significant changes. Relatively few studies have focused on the airflow and contaminant diffusion path caused by such internal partitions [13,14]. Bauman et al. conducted a full-scale measurement to study the effects of partitions on air movement and thermal comfort in office spaces, and concluded that the height of the partitions had a significant effect on thermal comfort under mixed-ventilation mode [15]. Lee et al. also predicted indoor air quality and ventilation performance with internal partitioning based on a small model test chamber and computational fluid dynamics (CFD) technology under mixed ventilation [16,17].

The above studies clearly show that internal partitions have a great impact on ventilation performance. However, these works primarily focused on airflow patterns under mixed ventilation, while the effects of both contaminant source characteristics and the locations of supply and exhaust openings on airflow patterns and pollutant distributions were not considered [18,19,20,21]. Therefore, this paper implemented a numerical simulation of indoor airflow and contaminant distribution under different ventilation patterns to study the effects of internal partitions and the locations of supply and exhaust openings. The contaminant source characteristics were also taken into consideration during this study.

## 2. Validation of the CFD Model

### 2.1. Model Test

**Figure 1:**
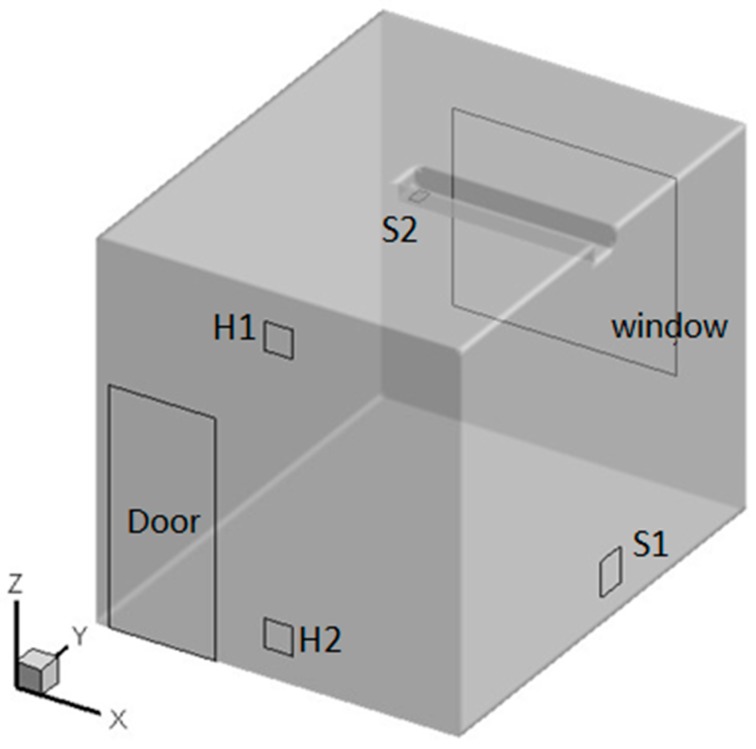
Schematic diagram of the test chamber.

In order to have full confidence in the simulated results, an airflow distribution experiment in a single chamber was designed. The size of the environmental chamber was 3.7 × 3.0 × 3.0 m^3^. It had seven ventilation openings located at the cell and the side walls, as illustrated in Figure 1, which could simulate various ventilation patterns by employing different combinations of the openings. The field measurement was carried out under down-supply up-return (S1H1, case A) and top-supply down-return (S2H2, case B) ventilation patterns. The coordinates of the central points of each opening were: H1 (1.5, 0, 0.36), H2 (1.5, 0, 2.62), S1 (3, 1.5, 0.36), and S2 (1.5, 1.8, 3). The numerical simulation results were compared and verified with the experimental data.

**Figure 2:**
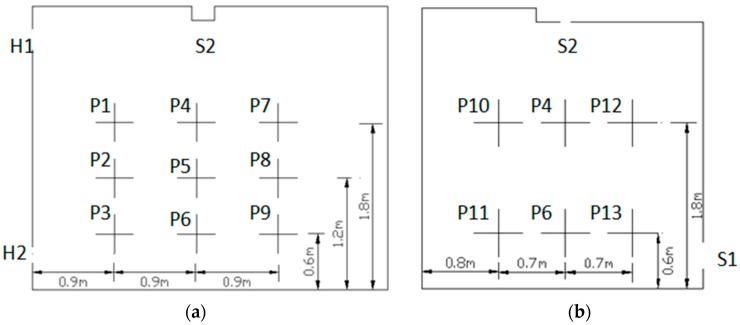
Detailed locations of the measurement points: (**a**) Plane X = 1.5 m; (**b**) plane Y = 1.8 m.

In order to ensure approximately the same boundary conditions between the experiment and the simulation, both air supply volumes and air velocities at supply openings were measured and compared to identify the supply air velocity in the simulations. Based on the instructions of ASHRAE (American Society of Heating, Refrigerating, and Air-Conditioning Engineers) standards and other literature, 13 measurement points were selected [22,23]. These sampling points were 0.6 m, 1.2 m, and 1.8 m away from the ground, as illustrated in Figure 2. During the experiment, the gaps in each joint and other unused openings were filled with sealing material to minimize air leakage. The ventilation system was turned on 30 min before the measurement began. The instrument measurement range and accuracy are shown in Table 1. In order to minimize measurement uncertainty, an analysis was performed beforehand to identify the suitable averaging time. Then, all the data were obtained by a 25 min average, which can ensure acceptable repeatability in the results.

### 2.2. Comparisons between Simulations and Measurements 

**Figure 3:**
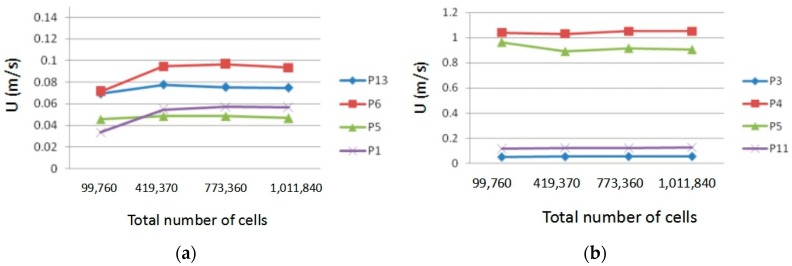
Results of grid independent test. (**a**) Case A; (**b**) case B.

The numerical simulations were carried out by Ansys Fluent commercial CFD software, using the same configurations as the physical experiments. A second-order upwind scheme and the SIMPLE algorithm were employed. RNG (renormalization group) k-epsilon with a standard wall function was used to solve the indoor airflow, with the model constants $C_{1\varepsilon }^{\mathrm{RNG}}$ = 1.42 and $C_{2\varepsilon }^{\mathrm{RNG}}$ = 1.68. Standard κ−ε and RNG κ−ε turbulence models are two widely used models for predicting turbulent airflow. The equations of both standard κ−ε and RNG κ−ε turbulence models can be easily found in the related references, which are not listed here. Compared with the standard κ−ε model, the RNG κ−ε model allows better handling of near-wall flows with high accuracy, and has been widely used in indoor airflow simulation. The difference between the standard and RNG κ−ε models is the calculation of turbulent viscosity. In the standard κ−ε model, $\mu_{t}$ is defined as $\mu_{t}=\rho C_{\mu }(k^{2}/\varepsilon )$, which denotes gas phase turbulent viscosity. In the RNG κ−ε model using a differential viscosity model, $\mu_{eff}$, which denotes effective viscosity, is calculated by:

(1) $$d\left(\frac{\rho^{2}k}{\sqrt{\varepsilon \mu }}\right)=1.72\frac{\hat{v}}{\sqrt{{\hat{v}}^{3}-1+C_{\nu }}}d\hat{v}$$

where $\hat{v}=\mu_{eff}/\mu$ and $C_{\nu }\approx 100$. Boussinesq approximation was used to take into account buoyancy effects induced by temperature differences. In the simulations, the mass fraction of the pollutant (Y) is predicted through the solution of the advection-diffusion equation. This module computes the diffusive mass flux ($\vec{J}$) which satisfies the conservation of mass as follows:

(2) $$\vec{J}=-D_{eff}\nabla Y$$

where $D_{eff}$ is the effective diffusion coefficient for the pollutant in the mixture. In turbulent flows, for standard and realizable κ−ε models, $D_{eff}=\rho \left(D_{m}+\frac{\mu_{t}}{Sc_{t}}\right)$, where ρ is the mixture density, and $D_{m}$ is the molecular diffusion coefficient for the pollutant in the mixture. $Sc_{t}=\mu_{t}/D_{t}$ is the turbulent Schmidt number and $D_{t}$ is the turbulent diffusivity of species. $Sc_{t}$ describes the relative diffusion of momentum and mass due to turbulence. $Sc_{t}$ is commonly set to 0.7 in standard and realizable κ−ε models. For the RNG κ−ε model, $D_{eff}=\alpha c_{p}\mu_{eff}$, and in the diffusion dominated region α is computed by the RNG theory:

(3) $${\left|\frac{\alpha -1.3929}{\alpha_{0}-1.3929}\right|}^{0.6321}{\left|\frac{\alpha +2.3929}{\alpha_{0}+2.3929}\right|}^{0.3679}=\frac{\mu_{\mathrm{mol}}}{\mu_{eff}}$$

where $\alpha_{0}=1/Sc$ (Sc is the molecular Schmidt number). In the fully turbulent region, α=1.393. The control equation discretization was executed by employing the finite volume method [24,25]. Structured grids were used in this study. The number of computational cells was 419,370, which was checked to guarantee grid independence, as shown in Figure 3.

**Figure 4:**
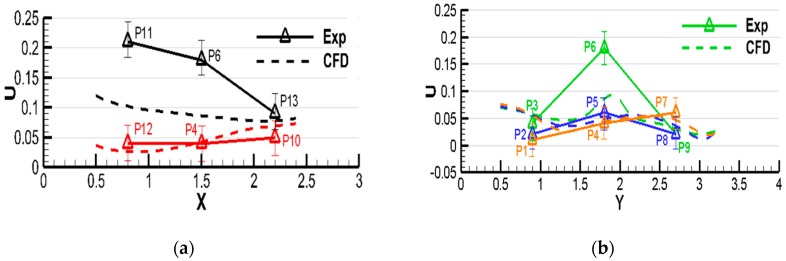
Comparison between experimental measurements and computational results for case A ((**a**): along the x-axis, (**b**): along the y-axis. The same color represents the same location, Exp: Experiment, CFD: computational fluid dynamics).

**Figure 5:**
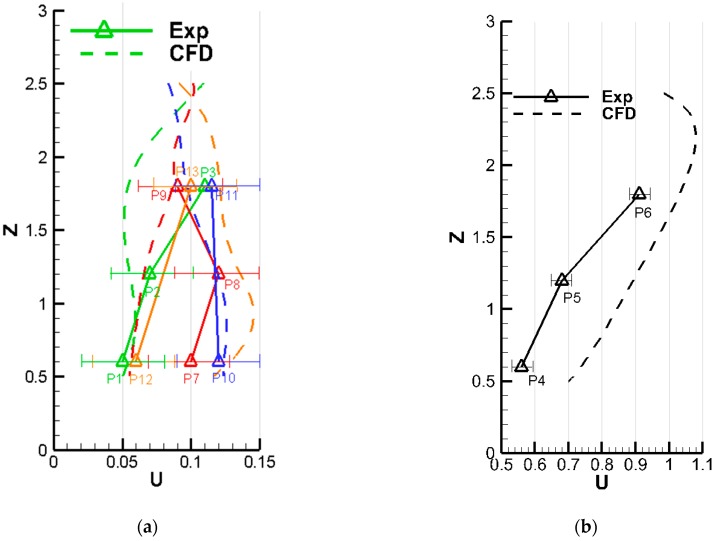
Comparison between experimental measurements and computational results for case B ((**a**) and (**b**) were along the z-axis, which passed different measurement points. The same color represents the same location, Exp: Experiment, CFD: computational fluid dynamics).

For the convenience of comparison, several poles that passed the measurement points shown in Figure 2 were selected for velocity comparisons, as shown in Figure 4 and Figure 5. In Figure 4a, the selected poles were along the *x*-axis, while the poles shown in Figure 4b were along the *y*-axis, which passed different measurement points. The poles selected for comparison in case B were in the vertical direction; the pole in the center of the room is presented in Figure 5b and the others are shown in Figure 5a. In both figures, the same color represents the same location. The dashed lines were the predicted results along different lines which passes the measurement points, while the triangle symbols represented the measured values. It can be intuitively seen that the simulated value is consistent with the experimental value under the two tested ventilation patterns in case A, except for some noticeable discrepancies appearing at P11 and P6 on pole 1 in Figure 3. The probable reason is that the wind speed was too low to avoid the measurement error under the down-supply up-return pattern; also, it was easy for the point far away from the air supply outlet to bring significant simulation errors. In case B, the wind speed of P4, P5, and P6 located at the seal was placed separately on a map, and some gaps were found, as illustrated in Figure 4. In spite of that, overall, the selected numerical method shows acceptable performance in predicting the indoor airflow under the presented ventilation patterns. Thus, the numerical method with the RNG κ−ε model was used in the following studies.

## 3. Case Studies 

Hereafter, the validated numerical method was used to investigate the internal partition effect on airflow and pollutant distributions. CO_2_ was selected as the tracer gas to simulate the indoor gas-phase pollutant. The case studies in this work were performed with two typical models, named Model I and Model II. Model I was set up as a benchmark case in order to discuss the basic influences induced by indoor partitions under different conditions, while Model II was more complicated and closer to a real-life situation.

### 3.1. Model I Setup 

**Figure 6:**
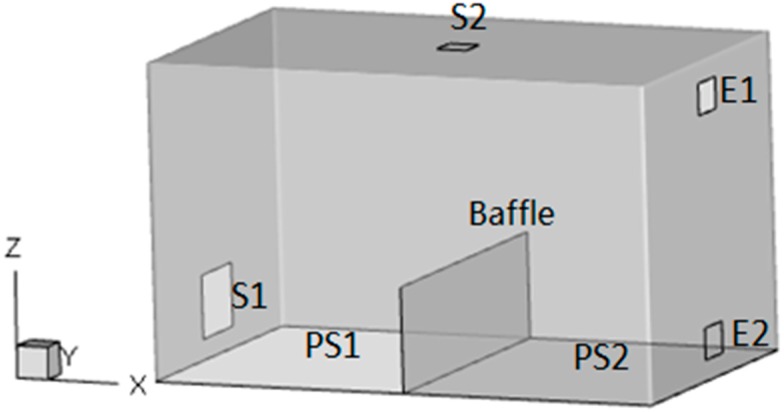
Physical Model I.

A simplified office room model was established, as presented in Figure 6. The specific size of the model was 5.0 m × 3.0 m × 3.0 m in the X–Y–Z three-dimensional system, and the vent position, etc., are also shown in the figure. A baffle was placed in the middle of the room in the X direction, which represents the partition wall. The specific parameters are presented in Table 2. The coordinates of the central point of each opening were: S1 (0, 1.5, 0.55) and S2 (2.5, 1.5, 3), E1 (5, 1.5, 2.6), and E2 (5, 1.5, 0.4).

The number of computational cells was 360,000, which was checked to guarantee grid independence. The pollutant release intensity was 4.25 μg/(m^2^·h). The ambient air temperature and supply temperature were 27 °C and 25 °C, respectively. Adiabatic wall conditions were applied for the internal partitions, ceiling, and floor. The air changes per hour (ACH) rate was 10 [26]. The detailed conditions for different cases are shown in Table 3.

### 3.2. Model II Setup 

**Figure 7:**
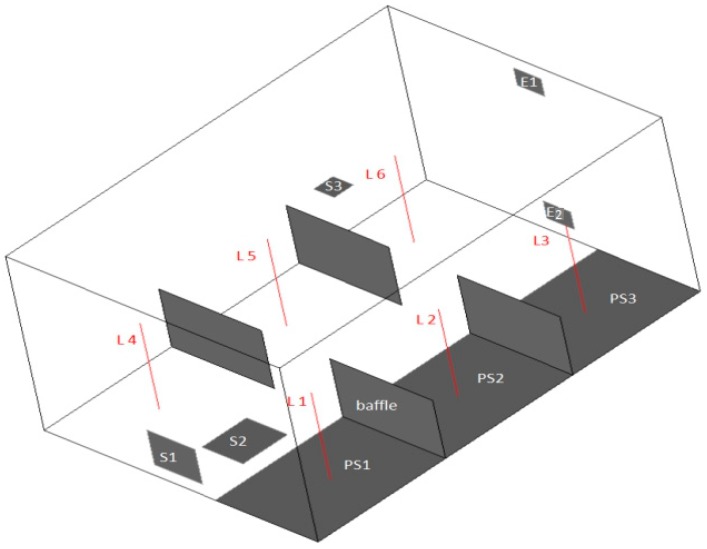
Physical Model II.

An office model with a size of 6 m (L) × 4 m (W) × 3 m (H) was established, which is close to a real-life situation. The office was divided into six even areas by 1.5 m (W) × 1 m (H) baffles with a 1 m wide walkway in the middle. Figure 7 illustrates the baffle and vent locations and pollution sources under the up-supply down-return, floor-supply up-return, and down-supply up-return ventilation patterns. In Table 4, the location and size are specified, and the coordinates of the central point of each opening were: S1 (0, 2, 0.6), S2 (1, 2, 0), S3 (3, 2, 3), E1 (6, 2, 2.65), and E2 (6, 2, 0.6). Tracer gas was CO_2_, with a release rate of 4.25 μg/(m^2^·h). The frequency of air change was set at 10 times per hour. The case setup is presented in Table 5.

## 4. Simulation Results and Analysis 

### 4.1. Airflow Patterns in Model I

Two commonly used ventilation modes, top-supply down-return and down-supply up-return, were selected for comparison. Case 1 and case 3 were selected to make a concrete analysis. 

**Figure 8:**
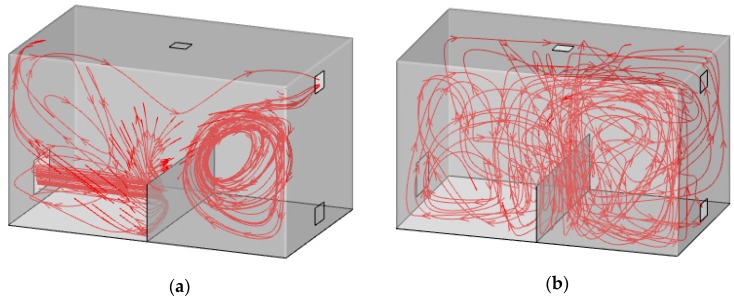
Streamlines under two ventilation patterns in Model I: (**a**) Down-supply up-return; (**b**) top-supply down-return.

As displayed in Figure 8a, the flow velocity flowed out in a direction parallel to the ground and changed its direction when encountering the baffle under the down-supply up-return ventilation pattern. Since the supply was located on one side of the partition, the blocking effect on the flow field was obvious. The air partially moved along the baffle until crossing it, produced a vortex in the right part, and finally moved out from the upper exhaust outlet. Therefore, strong disturbance existed in the left lower part and the right middle part of the room model, so that fresh air rolled up the contaminants in the left lower part and carried them to the right side. The airflow disturbance led to a dead zone, so that the indoor pollutants were distributed unevenly in the whole room. 

As shown in Figure 8b, the flow velocity moved vertically downward and spread to both sides when encountering the ground under the top-supply down-return ventilation pattern. The air partially flowed out from the exhaust outlet on the right side along the ground, and partially rolled up to generate a vortex. Airflow disturbance was extensive all over the space, which caused the indoor pollutants to be equally distributed in the whole room. Since the baffle was set in the middle of the room, it influenced the whole flow field slightly.

### 4.2. Pollution Concentration Distributions in Model I

**Figure 9:**
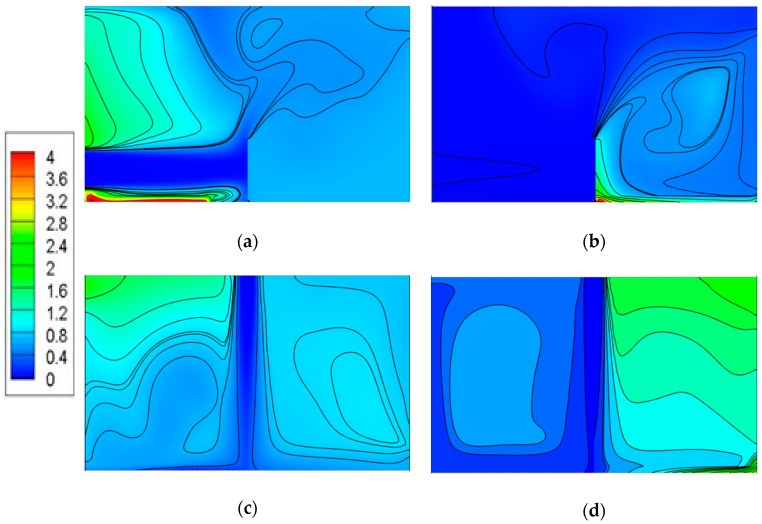
CO_2_ concentration cloud map of Y = 1.5 m section: (**a**) Case 1, (**b**) case 2, (**c**) case 3, (**d**) case 4.

Figure 9 shows the pollutant concentration distributions on a vertical plane (Y = 1.5 m) under different ventilation modes. The concentration values were normalized by $C_{1}=C/C_{e}$, in which $C_{e}$ is the facet-average concentration exhaust and C is the concentration at different positions, which can be obtained from the simulation results. For the down-supply up-return ventilation pattern, the pollution source was PS1 and PS2 in case 1 and case 2, respectively, as shown in Figure 9a,b. It is clearly seen that with the internal partition, the position of the pollution source could strongly affect the indoor pollutant distribution under this kind of ventilation pattern. When the source was located to the left side of the baffle, i.e., near the supply inlet, the pollutant concentration was relatively intense. This indicates that it was hard to discharge the pollutants that accumulated in this upper-left area because of the blocking effect induced by the internal partition. When the pollution source was located to the right side of the baffle, i.e., near the exhaust outlet, the pollutant concentration in the right part was relatively intense and the concentration level was much lower. 

For the top-supply down-return ventilation pattern, the pollution source was PS1 and PS2 in case 3 and case 4, respectively, as displayed in Figure 9c,d. When the pollution source was located to the left side of the baffle, the pollutant concentration was relatively high in the upper-left area and lower in the right part. On the contrary, when the pollution source was located to the right side of the baffle, the concentration in the right part was much higher than in the left part. This indicates that the top-supply ventilated mode can maintain concentration stability in both the left and right areas divided by the baffle and block the mutual diffusion of contaminants between the two areas when the pollution source is close to the exhaust outlet. When the source was located at the supply inlet, the pollutants would diffuse between the two areas to a certain degree.

**Figure 10:**
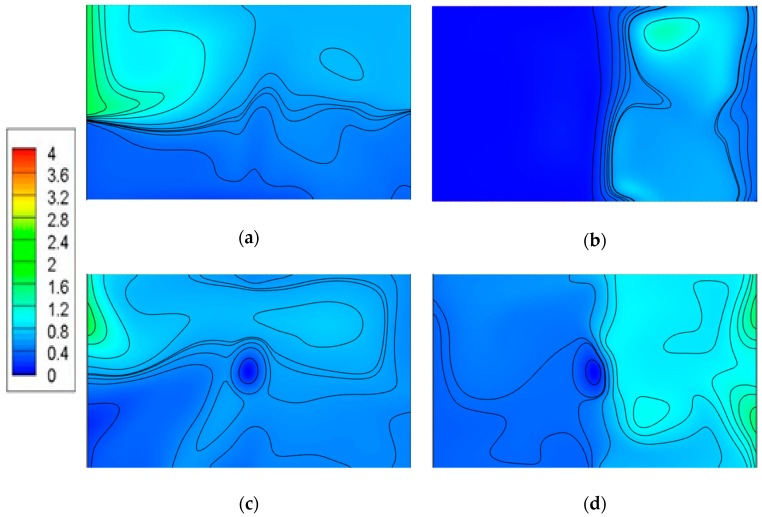
CO_2_ concentration cloud map of Z = 1.6 m section: (**a**) Case 1, (**b**) case 2, (**c**) case 3, (**d**) case 4.

Figure 10 shows the pollutant concentration distributions on a horizontal plane (Z = 1.6 m) under different ventilation modes. For the down-supply up-return ventilation pattern, the pollution source was PS1 and PS2 in case 1 and case 2, respectively, as shown in Figure 10a,b. Figure 10a shows that the contaminant concentration in the left area was comparatively high and a small fraction of the contaminant was brought to the right area due to the carrying effect of the air supply, resulting in the presence of CO_2_ in the right area. Figure 10b clearly shows that the pollutants were accumulated in the right area and the concentration in the left area is extremely low. The results imply that the fresh air flow near the supply inlet has little impact on the contaminant diffusion when the pollution source is located near the exhaust in a partitioned zone under this kind of ventilation pattern. Conversely, when the source is near the supply, the fresh air moving to the right area can influence the diffusion. 

The concentration distribution shown in Figure 10c features low concentration and even distribution, while in Figure 10d, it is uneven, with left-low and right-high and a higher surface-average concentration in this plane, indicating that the top-supply down-return ventilation works worst if the pollution source is PS2 for the pollutant distribution in this section.

### 4.3. Pollution Removal Efficiency in Model I 

For further study on the effect induced by the internal partition, additional cases were simulated with changes in the baffle height. The pollutant removal efficiency was used to evaluate the ventilation effectiveness under different scenarios. It was defined as $\varepsilon =\left(C_{e}-C_{s}\right)/\left(\bar{C}-C_{s}\right)$, in which $C_{s}$ and $C_{e}$ were the facet-average concentrations at supply and exhaust, respectively, and $\bar{C}$ was the volume-average concentration in the test room, which can be derived from the simulation results. 

**Figure 11:**
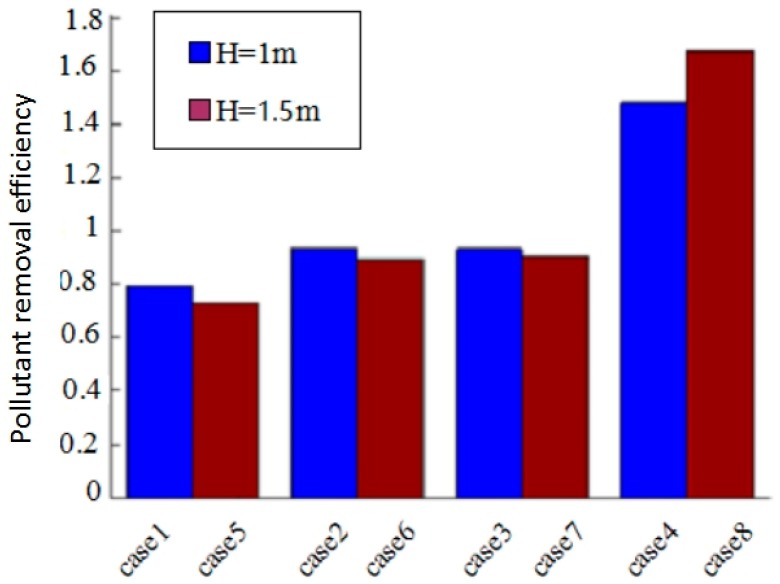
Pollutant removal efficiency under different scenarios.

Figure 11 shows the pollutant removal efficiency under different scenarios, in which the cases with different baffle heights are put together for direct comparison. The results show that the effect of the baffle height is not significant with regard to the **ε** value under the same ventilation mode and pollutant source location, and the differences between pairs of cases were generally about 10%. The ventilation mode and pollutant source location play dominant roles. The highest **ε** value is around 1.65 in case 8, while the lowest is around 0.75 in case 5. In case 8, the supply velocity is relatively high under the top-supply down-return ventilation mode, and the higher baffle restricts the contaminant to the right zone near the exhaust, which can be easily discharged compared with the other cases. Thus, the pollutant removal efficiency is the highest among all the cases.

### 4.4. Velocity Distributions along Vertical Lines in Model II

**Figure 12:**
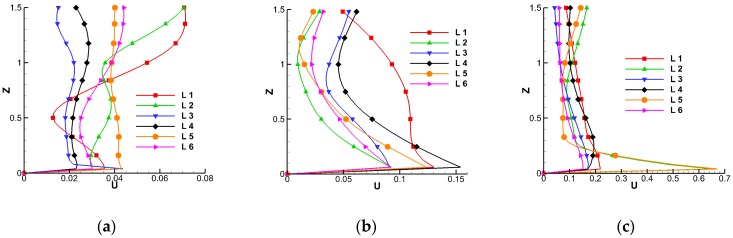
Velocity distributions along different lines in the center of subzones in Model II: (**a**) Down-supply up-return; (**b**) floor-supply up-return; (**c**) top-supply down-return.

In the middle of each zone, a line 1.5 m tall, representing the height of a sitting person, was selected for velocity analysis, as shown in Figure 12. Under A and B ventilation modes, when the supply air velocity was relatively low, the velocity profile along different lines was less affected by the air-supply mode, as shown in Figure 12a,b. It should be noted that both A and B ventilation modes are commonly used under conditions when there is temperature stratification in the indoor environment. Thus, the flow field affected by the buoyancy effect should be taken into consideration, which is out of the scope of our work. Under top-supply mode, the indoor air was well mixed, and the wind speed reached the highest in the air-supply position, as illustrated in Figure 12c. 

### 4.5. Pollutant Concentration Distributions in Model II

**Figure 13:**
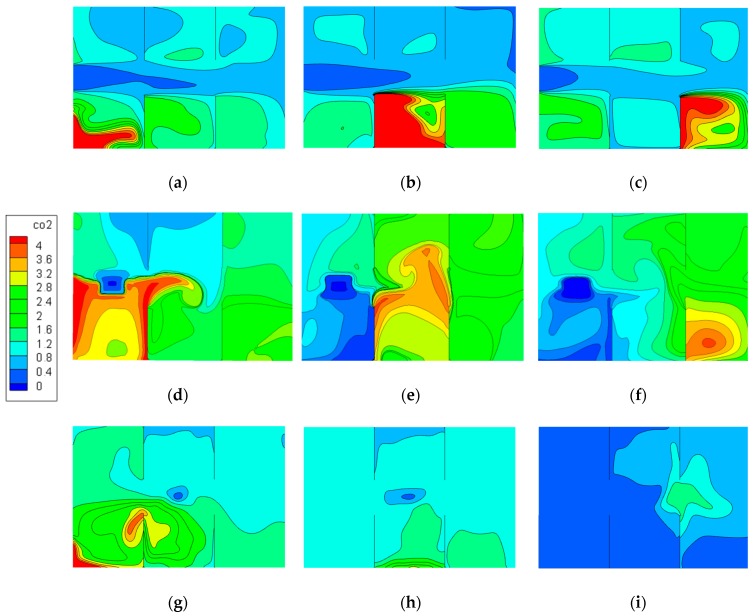
Distribution of CO_2_ concentration at the height Z = 0.75 m: (**a**) Case 9, (**b**) case 10, (**c**) case 11, (**d**) case 12, (**e**) case 13, (**f**) case 14, (**g**) case 15, (**h**) case 16, (**i**) case 17.

Figure 13 shows the pollutant concentration distributions on a horizontal plane (Z = 0.75 m) under different ventilation modes and pollutant source locations. This plane represents the breathing zone while occupants sit quietly. CO_2_ concentration is in dimensionless form, as presented in Figure 9 and Figure 10. As shown in the figures, cases in the same row adopted the same ventilation pattern, and the pollution source was located in the same position for cases in the same column.

Overall, the results indicate that the top-supply down-return ventilation pattern is more conducive to pollutant dispersion than the floor-supply up-return and down-supply up-return patterns in this model; consequently, the overall concentration of contaminants is relatively lower and pollutants are distributed uniformly in the whole area, which was the same trend as revealed under Model I.

Figure 13a–c illustrates three cases under the down-supply up-return ventilation pattern with different pollutant source locations. As seen in the figure, the baffles had a great impact on pollutant distribution under this pattern. The pollution source in Figure 13a was near the supply inlet, and pollutants at the corners of walls could almost not be expelled, but the concentration close to the walkway was comparatively low because of the air supply. The source in Figure 13b is in the middle area, where the air change performance was extremely poor due to baffles on both sides, and pollutants were accumulated near the left side of the baffle and the wall. The source in Figure 13c was close to the exhaust outlet and the concentration level near the baffle was high, but the concentration in the partition zone where the pollution source was located was lower compared with the previous two cases. The above results show that pollutants can accumulate in the source zone under this ventilation pattern, which has a certain influence on the same partition zone, while the concentration levels in the partitions on the other side of the walkway were comparatively lower.

Figure 13d–f shows three cases of the floor-supply up-return ventilation pattern with different pollutant source locations. Compared with the down-supply up-return pattern, the blocking effect of the baffles on pollutant dispersion was slight. As shown in Figure 13d, the pollutants mainly concentrated around the wall and baffle of the pollution source zone because of the obstruction effect of the baffle. When the pollution source was located at PS2, it showed the same trend as case 12, and the contamination also mainly gathered on the side of the baffle, as illustrated in Figure 13e. The source in Figure 13f was close to the exhaust outlet. It is easy to see that the pollution gathered in the middle of the zone. The air supply had a great influence on the diffusion of pollutants. As the pollution source was far away from the air outlet, the accumulation of pollutants gradually slowed down.

Figure 13g–i illustrates three cases under the top-supply down-return ventilation pattern, indicating that the blocking effect caused by the baffle was relatively slight, thus the contaminant concentration in the pollution source zone was high while concentration was distributed uniformly in the whole room. As displayed in Figure 13g, only when the source was away from the outlet were contaminants at the corners of the room easier to gather. Pollutants were more likely to diffuse to other zones under this ventilation pattern. The overall concentration was the lowest when the source was close to the exhaust outlet.

The above case setup simulates working partition zones in a real office. The simulation results under different ventilation patterns further demonstrate the influence of baffles and the pollution source location on indoor air and contaminant distribution.

## 5. Discussion

Based on the comparison between experiments and numerical simulations, a validated CFD method was used to simulate airflow patterns and pollutant distributions with different pollution sources under two typical air supply and ventilation modes in two office layouts. The effect of the internal partition height was also taken into consideration.

The simulation results show that baffles will lead to great changes in the indoor airflow distribution, which is not conducive to improving the indoor air quality, so obstacles such as baffles should be designed carefully based on the existing ventilation strategy. The source position still plays an important role in the distribution of indoor pollutants.

When there is a baffle in the room, the location of the pollution source has a larger impact on the down-supply up-return ventilation mode than the floor-supply up-return and top-supply down-return modes. However, the down-supply up-return ventilation mode works better than the other modes for the entire pollution distribution. Under the same ventilation mode and pollutant source, the effect of the baffle’s height is not significant with regard to the pollutant removal efficiency. The presented results could be helpful for occupants to design internal partitions in a large office. Further work needs to be done on the effect of the position of the partitions on the performance of different ventilation modes, and the office layout is another key factor. It would be better if we took dispersion measurements and compared them with the simulation results in further investigations, which could make the simulation results more reliable. It should be noted that CO_2_ was employed as a gas tracer to illustrate the air contaminant distribution features in this study; the detailed dispersion characteristics can be influenced by the physical conditions of the air pollutants, such as density, etc., which attention should be paid to in further studies.

## 6. Conclusions

The present work numerically evaluated the effect of internal partitions on airflow and concentration distribution under three ventilation strategies. The results show that different ventilation modes have great influence on indoor airflow distribution, and the top-supply down-return ventilation mode is better than the down-supply up-return and flow-supply up-return modes at providing a desirable airflow pattern. For layouts using baffles to separate different zones, the baffles will significantly affect the indoor airflow distribution. It is suggested that the baffles’ locations should be carefully designed in order to avoid blocking the airflow. It would be better to make the air flow over or fall on the upper part of the obstacle. Additionally, the effect of pollutant locations was also examined under different ventilation modes. When indoor pollution sources exist, the top-supply down-return ventilation mode shows better performance in removing pollutants. It should be noted that both down-supply and floor-supply ventilation modes are commonly used under the condition of temperature stratification in an indoor environment, and the effect of temperature plays a key role when assessing indoor airflow and dispersion problems. It should be noticed that CO_2_ was used as a tracer gas in this study, which has an obvious difference in density compared with air. The buoyancy effect induced by the density difference cannot be overlooked. In addition, both down-supply and floor-supply ventilation modes are commonly used under the condition of temperature stratification in an indoor environment, and the effect of temperature plays a key role when assessing indoor airflow and dispersion problems. Thus, the flow field affected by the buoyancy effect should be taken into consideration when there is a noticeable temperature difference, which is out of the scope of our work. The findings of this study can be helpful for both ventilation design and interior design in modern offices with large open spaces.

## Figures and Tables

**Table 1:** Test items and instruments.

Test Item	Instrument Name	Measurement Range	Accuracy	Sample Interval
Air velocity	Alnor Balometer Capture Hood EBT731	Air volume: 42–4250 m^3^/h Air velocity: 0.125–40 m/s	0.1 m^3^/h	10–600 s
Indoor velocity field/temperature field	Swema 03+ omnidirectional anemometer	Air temperature: +10 to +40 °C Air velocity: 0.05–5.00 m/s	0.03 m/s/3%	0.2 s

**Table 2:** Physical parameters of Model I.

Name	Room	Supply Inlet S1	Supply Inlet S2	Exhaust Outlet E1	Exhaust Outlet E2	Baffle	Pollution Source PS1	Pollution Source PS2
Size	5.0 m × 3.0 m × 3.0 m	0.7 m × 0.6 m	0.3 m × 0.3 m	0.4 m × 0.3 m	0.4 m × 0.3 m	3 m × 1.0 m	2.5 m × 3.0 m	2.5 m × 3.0 m

**Table 3:** Case setup (Model I).

Case No.	Inlet	Outlet	Air Velocity (m/s)	Air Change Rate (times/h)	Pollution Source	Baffle Height (m)
Case 1	S1	E1	0.3	10	PS1	1.0
Case 2	S1	E1	0.3	10	PS2	1.0
Case 3	S2	E2	1.4	10	PS1	1.0
Case 4	S2	E2	1.4	10	PS2	1.0
Case 5	S1	E1	0.3	10	PS1	1.5
Case 6	S1	E1	0.3	10	PS2	1.5

**Table 4:** Physical parameters of Model II.

Name	Room	Supply Inlet S1	Supply Inlet S2	Supply Inlet S3	Exhaust Outlet E1	Exhaust Outlet E2	Baffle	Pollution Source PS1, PS2, PS3
Size	6.0 m × 4.0 m × 3.0 m	0.7 m × 0.6 m	0.7 m × 0.6 m	0.3 m × 0.3 m	0.4 m × 0.3 m	0.4 m × 0.3 m	1.5 m × 1 m	2 m × 1.5 m

**Table 5:** Case setup (Model II).

Case No.	Inlet	Outlet	Air Change Rate (times/h)	Pollution Source	Baffle Height (m)
Case 9	S1	E1	10	PS1	1
Case 10	S1	E1	10	PS2	1
Case 11	S1	E1	10	PS3	1
Case 12	S2	E1	10	PS1	1
Case 13	S2	E1	10	PS2	1
Case 14	S2	E1	10	PS3	1
Case 15	S3	E2	10	PS1	1
Case 16	S3	E2	10	PS2	1
Case 17	S3	E2	10	PS3	1
